# Perceived stress and diet quality in women of reproductive age: a systematic review and meta-analysis

**DOI:** 10.1186/s12937-020-00609-w

**Published:** 2020-08-28

**Authors:** Karim Khaled, Fotini Tsofliou, Vanora Hundley, Rebecca Helmreich, Orouba Almilaji

**Affiliations:** 1 Department of Rehabilitation & Sport Sciences, Faculty of Health & Social Sciences, Bournemouth University, Bournemouth, BH1 3LT UK; 2 Centre for Midwifery, Maternal & Perinatal Health, Faculty of Health & Social Sciences, Bournemouth University, Bournemouth, BH1 3LT UK; 3 Department of Graduate Studies, Cizik School of Nursing, University of Texas Health Science Center at Houston, Houston, USA; 4 Department of Medical Science and Public Health, Faculty of Health & Social Sciences, Bournemouth University, Bournemouth, BH1 3LT UK

**Keywords:** Diet quality, Diet, Stress, Women, Reproductive age, Systematic review, Meta-analysis

## Abstract

**Background:**

Poor diet quality is associated with obesity-related morbidity and mortality. Psychological stress can increase unhealthy dietary choices, but evidence pertinent to women of reproductive age remains unclear. This paper systematically reviewed the literature to determine the association between psychological stress and diet quality in women of reproductive age.

**Methods:**

Medline, CINAHL, Scopus, Cochrane Library, Web of Science, and Sciencedirect were searched. Data extraction was determined by the PEO. Inclusion criteria consisted of: English language, stress (exposure) measured in combination with diet quality (outcome), healthy women of reproductive age (18–49 years old (population)). Observational studies, due to the nature of the PEO, were included. Quality assessment used the Risk of Bias in Non-randomised Studies from the Cochrane Handbook of Systematic Reviews of Interventions. Meta-analysis was conducted using random-effect model to estimate the Fisher’s z transformed correlation between stress and diet quality with 95% confidence interval (CI).

**Results:**

From 139,552 hits, 471 papers were screened; 24 studies met the inclusion criteria and were conducted in different countries: 8 studies on diet quality and 16 on food intake and frequency of consumption. Studies of diet quality consisted of six cross-sectional and two longitudinal designs with a total of 3982 participants. Diet quality was measured with diverse indices; Alternate Healthy Eating Index (*n* = 2), Healthy Eating Index (n = 2), Dietary Approach to Stop Hypertension (DASH) Diet Index (n = 2), Dietary Quality Index- Pregnancy (n = 2), and Dietary Guideline Adherence Index (*n* = 1). Most studies used Cohen’s perceived stress scale and no study measured biological stress response. After sensitivity analysis, only 5 studies (3471 participants) were included in the meta-analysis. Meta-analysis revealed a significant negative association between stress and diet quality with substantial heterogeneity between studies (r = − 0.35, 95% CI [− 0.56; − 0.15], *p* value < 0.001, Cochran Q test *P* < 0.0001, I^2^ = 93%).

The 16 studies of food intake and frequency of consumption were very heterogeneous in the outcome measure and were not included in the meta-analysis. These studies showed that stress was significantly associated with unhealthy dietary patterns (high in fat, sweets, salt, and fast food and low in fruits, vegetables, fish, and unsaturated fats).

**Conclusion:**

Future studies that explore diet quality/patterns should include both diet indices and factor analysis and measure biological markers of stress and dietary patterns simultaneously.

## Background

The rate of obesity has increased alarmingly in the past twenty years across all age groups, especially among young adults [1]. In women of reproductive age, obesity is associated with type-2-diabetes, hypertension, decreased fertility and delayed conception, high birthweight and congenital anomalies [2–4]. These women are at increased risk of obesity related morbidity and mortality especially during pregnancy when metabolic complications might deteriorate and cause gestational diabetes, pre-eclampsia, miscarriage, and various cardiovascular disorders putting both the mother and baby at increased health risk [5]. Preventing weight gain in women of reproductive age through healthy diet is crucial and would benefit the next generation [6, 7]. Poor dietary patterns are major predictors of increased adiposity and a higher diet quality is associated with reduced risk of obesity-related metabolic disorders [6, 8]. Recently, diet patterns have been derived in nutrition epidemiological studies by measuring the whole diet instead of single nutrients [9]. Indeed, the overall food pattern is considered a more realistic approach to investigate the association between diseases and food consumption rather than single nutrients [9]. Diet patterns/quality can be estimated via a posteriori approach based on statistical methods such as factor analysis, or a priori- defined diet quality score which measures adherence to specific dietary pattern indices such as the Mediterranean Diet Index [10]. These healthy dietary patterns (e.g. Mediterranean diet) have been associated with decreased risk of cardiovascular disease, diabetes, cancer, and hypertension in women of reproductive age, and this is why they are used to measure diet patterns/quality in recent epidemiologic studies [11, 12].

There are several factors that might affect diet patterns/quality such as adiposity, smoking, age, income, educational level, race/ ethnicity, marital status, and psychological factors [13, 14]. Particularly, there has been a growing interest in the role of stress in relation to human health [15, 16]. Stress is defined as an individual’s perception, appraisal, and response to a stimulus exhibited by the surrounding environment [17], and it happens when the person’s adaptive capacity is surpassed by the stimuli and demands of the environment [18]. Stress has been associated with diet patterns in young adults, and the dietary responses to stress are individualized [19, 20]. For example, some reviews and longitudinal studies investigated the effects of stress on energy intake and have found that with high levels of stress, 40% of people eat more, 40% eat less, and 20% eat the same amount of food compared to that consumed in the absence of stress [21–23]. The variance in the response to stress might be due to the duration of exposure to stress, the type of stressor, and the variation in the level of hunger and satiety at the start of the studies [24]. For example, mild/chronic stressors (such as long-term poverty, unemployment, unhappy marriage, etc.) increase the desire for food intake and binge eating, while sever/acute stressors (such as an upcoming work deadline or exam) induce restriction of food intake [24]. It is fundamental in this context to understand the types of food that are consumed and restricted under stress in order to estimate its health consequences. In general, studies have reported that highly stressed participants tend to consume hyper-palatable foods that are high caloric, low nutrient-dense (e.g. butter, cream cheese, full-fat products), and high fat foods even when there is no hunger or bodily demand for food [25–27]. The effects of stress have been found to be exacerbated in obese (BMI > 30 kg/m^2^) compared to normal weight individuals because the former have higher insulin resistance than the latter and demonstrate significantly higher activation of brain reward regions when exposed to stress [24, 28].

Recent studies among young adults and university students have found that perceived stress is a serious contributor to low diet quality [29, 30]. The majority of these studies have focused on food groups (such as fat intake) as a result of stress, rather than assessing the diet quality (a priori/ a posteriori) [30–32]. For example, there is evidence that females (18–29 years old), who report high levels of perceived stress (measured through the 14-item perceived stress scale), consume more fat than non-stressed females as assessed by the Night Eating Questionnaire [30–32]. When fruits and vegetables consumption was assessed in women of reproductive age, perceived stress was found to significantly decrease their intake [15, 16, 33–36]. Studies that have examined stress and diet have been limited in their approach. Habhab et al. [31] assessed the association between perceived stress and diet in females of reproductive age and found that participants in the high stress group (given unsolvable Sudoku) consumed more fats and sweets (measured through the Emotional Eating subscale) than individuals in the low stress group (given easy Sudoku). However, the sample size was small (40 participants), baseline hunger status was not measured, and the assignment of participants to low or high stress groups might have by chance assigned stressed individuals to the high stress group. In a study by Barrington et al. [37], higher levels of perceived stress were associated with higher fast food consumption in young women. However, the study used non validated single item scale to measure fast food intake.

In summary, the picture regarding the association between stress and diet in women of reproductive age remains unclear. This has gained attention recently, especially that diet-related diseases have been trending over the past few years among these women and studying the factors that might affect diet (such as stress) became crucial. To our knowledge, this is the first review of the association between stress and dietary patterns/quality specifically in women of reproductive age. The aim of this systematic review is to critically appraise the current literature and identify whether women who exhibit higher levels of stress have a poorer diet pattern/quality than women who exhibit lower levels of stress.

## Methods

The Meta-analysis of Observational Studies in Epidemiology (MOOSE) was used to guide this systematic review [38]. The association between psychological stress and diet quality was examined using the PEO (Population, Exposure, and Outcome) model: Population (women aged 18–49 years old), Exposure (Psychological Stress), Outcome (Diet Quality/Patterns of women of reproductive age).

### Search strategy

A literature search was conducted in December 2019 in Medline complete, CINAHL Complete, Scopus, Cochrane Library, Web of Science, and Sciencedirect. These databases were searched using appropriate key words and index terms where the PEO (Population, Exposure, and Outcome) model framed the search process (Table 1 in Additional file 1). The key words were then combined by the EBSCO host operator AND/OR. The databases search was limited to human studies and English language articles published between 2000 and 2019. The search strategy (Title/Abstract) is demonstrated in Additional file 1.

**Table 1:** Characteristics extracted from the 24 included studies: BS (Breakfast skippers), BE (Breakfast eaters), CS (Cross-Sectional), LG (Longitudinal), y (years), m (months), FFQ (Food Frequency Questionnaire), WFR (Weigh food record), SES (Socioeconomic status), PA (Physical Activity), AM (Anthropometric measures), − (not reported)

Author, Year	Country	Age and Number of Participants	Study Design	Participants in Study	Dietary Assessment Tool	Confounding Factors Identified
*8 studies on Diet Quality*						
*Richardson* et al. *2015* [39]	USA	18–44 y, *N* = 101	CS	Women who had a child up to age 5	24-h Dietary recalls	SES, AM
*Ferranti* et al. *2013* [10]	USA	Mean age 48 y, *N* = 433	LG (5 y follow up)	University and health center employees	FFQ	SES, PA, AM,
*Isasi* et al. *2015* [40]	USA	18–74 y, *N* = 3141	LG (9 m follow up)	Hispanic/Latino males and females	24-h Dietary recalls	SES, PA, AM
*El Ansari* et al. *2015* [41]	Egypt	16–30 y, *N* = 1483	CS	Undergraduate students males and females	FFQ	SES, PA, AM
*Valipour* et al. *2017* [42]	Iran	28–45 years old, *N* = 2134	CS	General Adults	FFQ	SES, PA, AM
*Fowles* et al. *2012* [43]	USA	Mean age 24.7 y, *N* = 71	CS	Low income pregnant women	24-h Dietary recalls	SES, AM
*Fowles* et al. *2011* [44]	USA	Mean age 25 y, *N* = 118	CS	Low income pregnant women	24-h Dietary recalls	SES, AM
*Widaman* et al. *2016* [45]	USA	Mean Age 25.1, *N* = 35 (BS) Mean Age 24.1, *N* = 40 (BE)	CS	Female habitual breakfast eaters and breakfast skippers	24-h Dietary recalls	PA, AM
*16 studies on Food Intake and Frequency of Consumption*						
*Vidal* et al. *2018* [1]	Peru	Mean Age: 19 y, *N* = 272	CS	Undergraduate students	Block fat screener	SES
*Nastaskin* et al. *2015* [46]	Canada	Mean age: 20 y, *N* = 113	CS	Students	Block fat screener/ Block sodium screener	SES, AM
*Pettit* et al. *2011* [47]	USA	18–24 y, *N* = 78	CS	Undergraduate students	Energy drink intake questions	SES
*Mikolajczyk* et al. *2009* [34]	Germany, Poland, Bulgaria	Mean age: 20 y, *N* = 1201	CS	Fist year undergraduate students	FFQ	–
*Errisuriz* et al. *2016* [48]	USA	Mean age: 18.9 y, N = 433	CS	Freshman students	Food and beverage frequency questions	SES, AM
*El Ansari* et al. *2014* [15]	UK	Mean age: 24.9 y, *N* = 2699	CS	Students	FFQ	–
*Ng* et al. *2003* [49]	USA	Mean age: 40 y, *N* = 6620	CS	Working adults	Block Fat Screener/ Alcohol frequency questions	SES, PA
*Barrington* et al. *2012* [37]	USA	18–65 y, *N* = 357	CS	Working adults	Single-item question for fast food intake/ 5-A-Day fruit & vegetable assessment tool	SES, PA, AM
*Grossniklaus* et al. *2010* [50]	USA	Mean age: 41.3 y, *N* = 64	CS	Working adults	3-day WFR	SES, AM
*Papier* et al. *2015* [16]	Australia	Mean Age 21.2 y, *N* = 397	CS	Students	FFQ	SES, PA, AM
*Roohafza* et al. *2013* [35]	Iran	Mean age: 38.4–39.5 y, *N* = 9549	CS	General adults	FFQ	SES, PA, AM
*Gonzalez* et al. *2013* [51]	Puerto Rico	21–30 y, *N* = 186	CS	First and second year students	Alcohol frequency questions	SES
*Tseng* et al. *2011* [36]	USA	Mean age 43.9 y, *N* = 426	CS	Premenopausal women	48- h Dietary recalls	SES
*Hinote* et al. *2009* [33]	8 post-Soviet republics	> 18 y, N = 10,454	CS	General adults	Questions about frequency of consumption	SES
*Hwang* et al. *2010* [52]	Korea	Mean age: 23.7 y, *N* = 570	CS	Vietnamese female marriage immigrants	1-day Dietary recalls	SES, PA, AM
*Wardle* et al. *2000* [53]	UK	Mean Age: 36.29 y, *N* = 58	CS	Staff of a store	24-h Dietary recalls	SES, AM

Alongside title and abstract searching, Medical subject headings (MeSH) were used when searching MEDLINE and CINAHL subject headings when searching CINAHL. The key terms used were: “psychological stress” AND “Diet”. Additionally, reference lists were checked, and authors of unpublished papers were contacted by email.

### Selection of studies

The reviewer (KK) screened the full texts of all potentially relevant papers, including those over which there was doubt, with excluded articles also reviewed by the second reviewer (FT) to ensure that studies are not erroneously excluded. Any disagreements were resolved by discussion, or arbitrated if necessary, by a third reviewer (VH). Similarly, if eligibility was unclear, this was discussed across the wider team (KK, FT, and VH).

### Inclusion and exclusion criteria

Studies were included in the review if they: i) enrolled healthy women aged 18–49 years old, ii) measured psychological stress (subjective and/or objective) as an exposure in combination with diet, iii) comprised observational quantitative studies looking at the association between stress and diet quality, iv) were in English language. Due to the limited resources available, it was not possible to translate non-English papers.

For studies in which the sample’s age range may in part be below or over the specified age range for this review, they were included if the mean age of the sample was between the age range of 18–49 years.

Articles were excluded if they: i) used qualitative methods, ii) enrolled exclusively men or participants with mean age outside the age range of 18–49 years old; iii) did not report stress data in a format that could be extracted; iv) comprised study sample with health conditions that may confound the diet stress relationship (e.g. depression, mental disorders, heart disease, diabetes, cancer, coeliac disease, eating disorders). Abstracts and unpublished studies were not included in this systematic review.

### Data extraction

Data extraction and coding stages of the review were completed by the first reviewer (KK) using structured data extraction forms. The following information was extracted from the manuscripts: first author, year of publication, location, study design, number of subjects, period of enrolment and follow-up, age, the exposure (self-reported stress measured via validated stress scales and/or via biological marker (e.g. cortisol levels in blood, hair or saliva)). A proportion of the extracted data (30%) was checked for accuracy by second reviewer (FT).

For the purpose of meta-analysis, a dataset containing the 7 studies [39–45] that initially qualified for meta-analysis was built. Ferranti et al. [10] was not among these studies as it did not report any effect size and hence should not be qualified for meta-analysis. The dataset was developed with the help of reviewer (OA) and included the following information from the studies: effect size, number of participants, first author surname, and year of publication. When only β coefficient was reported in any study, a proper conversion was carried out to transform β coefficient to correlation coefficient “r”. This was undertaken using the formula of imputing r value from β [54]: r = 0.98 β + 0.05 λ (restricted only to linear models and β values between ±0.5), where λ is an indicator variable that equals 1 when β is nonnegative and 0 when β is negative [54]. In the study by Richardson et al. [39]: r = 0.98 (− 0.18) + 0.05 (0) = − 0.1764. The β coefficient in Isasi et al. [40] is not within the exact range (± 0.5), however due to the large sample size in the study and the proximity of its β coefficient value to the range in the formula of imputing r from β, the formula was applied as follows: r = 0.98 (− 0.61) + 0.05 (0) = − 0.5978. The formula was not applied to Valipour et al. [42] as it is based on categorical dependent variable model, so this study was also excluded from the meta-analysis.

#### Study outcomes

Study outcomes included: dietary components (e.g. fat intake, alcohol intake, healthy versus unhealthy diet patterns) or adherence to diet indices (e.g. Alternate Healthy Eating Index (AHEI), the Dietary Approaches to Stopping Hypertension (DASH), and the Mediterranean Diet Score (MDS)).

### Quality evaluation

The first and second reviewers (KK, FT) assessed bias in all eligible studies using the Risk of Bias in Non-randomised Studies [55], which is recommended by the Cochrane Handbook of Systematic Reviews of Interventions [56]. The bias domains included in the quality assessments were bias due to confounding, bias in selection of participants, bias in classification of interventions, bias due to deviations from intended interventions, bias due to missing data, bias in measurement of outcome, bias in selection of the reported results. Any conflicting opinion of quality of studies was discussed with the third reviewer (VH).

### Meta-analysis

Meta-analysis was performed based on the Cochrane Handbook for Systematic Reviews of Interventions and Borenstein book on meta-analysis [57, 58]. Fisher’s z transformation of correlation was used as a summary measure of the association between diet quality and stress, whereby correlation coefficients were converted to Fisher’s z scale. Due to heterogeneity of the studies, particularly with respect to studies’ participants and the methods of measuring the exposure and the outcome, a random effect model has been applied for the meta-analysis. Higgin’s & Thompson’s I^2^ and Cochran’s *Q* measures were used to assess the between-study heterogeneity [58]. Outliers and influential studies were detected by identifying any study with a confidence interval that did not overlap with the confidence interval of the pooled effect through Baujat plot [57]**.** Publication bias was assessed through a Funnel plot. Sensitivity analysis was performed by applying trim and fill method [57, 58]. Following the Cochrane Handbook recommendations, a risk-of-bias assessment was performed for all included studies by creating a “weighted bar” which plots the distribution of risk-of-bias judgements within each bias domain. The figure was formatted according to the risk-of-bias assessment tool (ROBINS-I).

## Results

**Fig. 1:**
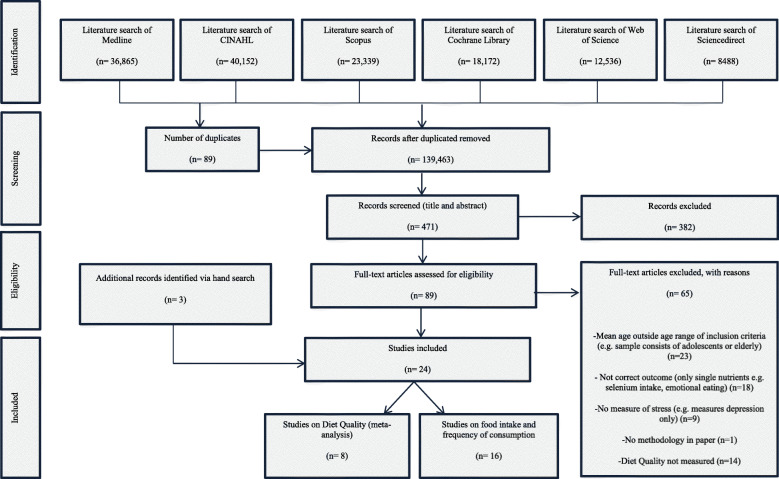
Meta-analysis of Observational Studies in Epidemiology (MOOSE) flow chart

The databases identified 139,552 hits; only 471 had a relevant title (Fig. 1; (MOOSE Checklist in Additional file 2). The titles and abstracts of these articles were screened further and 382 were deemed not relevant which yielded 89 articles for full-text screening. A further 65 studies were subsequently excluded as they did not meet the criteria. Three studies were eliminated after quality assessment for the following reasons: one study did not have a methods section [59] and two studies measured the emotional/psychological domain of eating as an outcome (disordered eating/emotional eating) [60, 61]. A total of 24 studies were included in the review: 8 studies on diet quality (measured the adherence to specific dietary indices as outcome) and 16 studies on food intake and frequency of consumption which reported consumption of different food components and nutrients as proxy measure for dietary patterns (Tables 1, 2, and 3 in Additional files 3, 4 and 5 respectively).

**Table 2:** Data values extracted from the included eight studies on Diet Quality: β (Beta coefficients), r (correlation coefficient), OR (Odd Ratio), ↑ (increase), ↓ (decrease), <= > (no association)

Author, Year	Stress Assessment Tool	Diet Quality Index	Association between Stress and Diet Quality	β coefficient, r, or OR
*Richardson* et al. *2015* [39]	- 14-item Perceived Stress Scale	- Healthy Eating Index 2010	<=>	β = − 0.18 (S.E 0.10, *p* = 0.08)
*Ferranti* et al. *2013* [10]	- 14-item Perceived Stress Scale - Beck Depression Inventory II	- Alternate Healthy Eating Index - Mediterranean Diet Index - Dietary Approach to Stop Hypertension Index	<=>	Not reported
*Isasi* et al. *2015* [40]	- 10-item Perceived Stress Scale - 8-item Chronic stress burden	- Alternate Healthy Eating Index 2010	↓	β = − 0.61 (− 1.18 to − 0.03)
*El Ansari* et al. *2015* [41]	- 4-item Perceived Stress Scale	- Dietary Guideline Adherence Index	<=>	r = 0.00, *p* = 0.98 β = 0.00 (− 0.13 to 0.13)
*Valipour* et al. *2017* [42]	- 12-item General Health Questionnaire	- Dietary Approach to Stop Hypertension Index	<=>	OR: 1.02 (0.78–1.33)
*Fowles* et al. *2012* [43]	- Edinburgh Postnatal Depression Scale - Prenatal Psychosocial Profile-stress subscale	- Dietary Quality Index- Pregnancy	↓	r = −0.35, p is not reported
*Fowles* et al. *2011* [44]	- Edinburgh Postnatal Depression Scale - Prenatal Psychosocial Profile-stress subscale	- Dietary Quality Index- Pregnancy	↓	r = − 0.293, *p* < 0.01
*Widaman* et al. *2016* [45]	- Wheaton Chronic Stress Inventory	- Healthy Eating Index 2010	↓ in breakfast skippers <= > in breakfast eaters	Empty calories (r = − 0.392, *p* = 0.027) Empty calories (r = − 0.104, *p* = 0.53)

**Table 3:** Data values extracted from the included studies on food intake and frequency of consumption: ↑ (increase), ↓ (decrease), <= > (no association)

Author, Year	Stress Assessment Tool	Association between Stress and the measured Food intake and frequency of consumption	Values
*Vidal* et al. *2018* [1]	14-item Perceived Stress Scale	↑ Fat intake	*p* = 0.005
*Nastaskin* et al. *2015* [46]	14-item Perceived Stress Scale	↑ Fat intake	*r*=*.* 35, *p < 0.*01
		↑Sodium intake	*r*=*.* 23, *p = 0.*07
*Pettit* et al. *2011* [47]	14-item Perceived Stress Scale	↑ Energy Drink intake	*r*=*.* 235, *p < 0.*01
*Mikolajczyk* et al. *2009* [34]	14-item Perceived Stress Scale	↑ Sweets, cookies, snacks, fast food	*p* = 0.03
		↓ Fruits/vegetables	p < 0.01
*Errisuriz* et al. *2016* [48]	Perceived stress single item scale (0–10)	↑ Soda, coffee, energy drink, salty snack, sweet snack, frozen food, and fast food consumption	p < 0.05
*El Ansari* et al. *2014* [15]	4-item Perceived Stress Scale	↑ Sweets, cookies, snacks, fast food	*P* = 0.017
		↓ Fruits and vegetables	*P* = 0.002
*Ng* et al. *2003* [49]	4-item Perceived Stress Scale	↑ High Fat diet	p < 0.01
		<= > Alcohol intake	p = 0.4
*Barrington* et al. *2012* [37]	10-item Perceived Stress Scale	↑ Fast food intake	z = 3.00, *P* = .003
		↓ Fruits and vegetables intake	z = − 3.01, P = .003
*Grossniklaus* et al. *2010* [50]	Perceived Stress Scale	<= > food and beverage intake	*p* > 0.05
*Papier* et al. *2015* [16]	Depression Anxiety Stress Scale (DASS)	↑ processed foods	p < 0.01
		↓ meat alternatives	*p* < 0.05
		↓vegetables and fruits	p < 0.01
*Roohafza* et al. *2013* [35]	-A12-item General Health Questionnaire (GHQ-12)	↑ Saturated oils	p < 0.01
		↓ Unsaturated oils	p < 0.01
		↓ Fruits	p < 0.01
		↓ Vegetables	p = 0.02
		↓ Meat	*p* = 0.03
		↓ dairy products	p < 0.01
*Gonzalez* et al. *2013* [51]	Cognitivist Systemic Model Academic Stress scale	↑ Alcohol intake	p < 0.05
*Tseng* et al. *2011* [36]	Migration–Acculturation Stressor Scale	↑ Energy density	-(β = 0.002, *p* = 0.04)
		↑ % energy from fat	-(β = 0.06, *p* = 0.05)
		↓ total grams of grains	-(β = −11.3, p < 0.0001)
		↓ Overall grain intake	-(β = −0.18, p = 0.03)
*Hinote* et al. *2009* [33]	12-item distress scale	↓ Meat, fish, vegetables, fruits, animal fat	p < 0.001
*Hwang* et al. *2010* [52]	Psychological Well-Being Index	↓ energy intake	-*p* = 0.011
		↓ carbohydrates	-*p* = 0.004
		↓ protein	-p = 0.021
		↓ fat	-*p* = 0.021
		↓ calcium	-*p* = 0.042
		↓ vitamin A	-*p* = 0.039
		↓ zinc	-p = 0.005
		↓ thiamine	-*p* = 0.006
		↓ riboflavin	-*p* = 0.013
		↓ folate	-p = 0.004
*Wardle* et al. *2000* [53]	10-item Perceived Stress Scale	↑ energy intake, ↑ saturated fats intake, ↑ fat intake	p < 0.05, *p* < 0.01, p < 0.05

### Characteristics of included studies

Two out of the eight studies that assessed diet quality were longitudinal cohort studies: [10] included 5 years of follow-up (*n* = 429), while [40] followed participants for 9 months (*n* = 3141) (Table 1). Both studies investigated psychological stress via the Perceived Stress Scale (PSS) at baseline; however, diet quality was investigated through different methods: [10] used food frequency questionnaire at baseline while [40] used two 24-h dietary recalls. The other six studies were cross-sectional, published between 2011 and 2017, and included a total of 3982 participants [39, 41–45]. Only two out of the eight studies were conducted outside of the USA [41, 42]. Two studies included pregnant women of reproductive age who fall in the age range 19–49 years old [43, 44]. Four studies recruited females only (18–45 years old) [39, 43–45] while the other four recruited both males and females (16–74 years old) [10, 40–42].

The 16 studies on food intake and frequency of consumption did not assess diet quality, but instead measured the different food components and nutrients. As a result, the studies were very heterogeneous. Studies were all of a cross sectional design and published between 2000 and 2018. Six studies were conducted in USA, two in UK, and the remaining eight were conducted in other countries. Two studies took place in more than one country: Mikolajczyk et al. [34] was done in three European countries (Germany, Poland, Bulgaria) and Hinote et al. [33] was done in eight post-Soviet republics. In only two studies, participants were 100% females; the rest had both males and females with more than half of the participants were females in all of these studies. One study did not specify the percentage of females in its sample [35]. Mean age of participants was between 18.9 and 43.9 years and the number of female participants ranged from 52 to 10,454 per study.

### Findings of the studies

In four of the eight studies on diet quality, stress was not associated with diet quality [10, 39, 41, 42], while in another three studies; stress was significantly associated with poorer diet quality [42.40.41] (Table 2). Interestingly, one study found that stress was significantly associated with lower diet quality in breakfast skippers only while no association was found in breakfast eaters [45].

The three studies that reported β coefficients indicated mixed results; two found no association [10, 41] and one found poorer diet quality when individuals were stressed [40]. Studies that reported correlation coefficient “r” found negative association between stress and diet quality [43, 44], no association [41], and mixed results (negative association in breakfast skippers/no association in breakfast eaters) [45] as shown in Table 2.

The outcomes of the 16 studies on food intake and frequency of consumption were very heterogeneous and thus it was not possible to perform a meta-analysis (Table 3). All studies that assessed fat intake found that perceived stress was significantly associated with increased fat consumption [1, 36, 46, 49, 53]. Only Hwang et al. [52] reported a significant decrease in fat intake, along with decreased intake of energy, carbohydrates, protein, calcium, vitamin A, zinc, thiamine, riboflavin, and folate, as a result of high stress (*p* < 0.05). The intake of fruits, vegetables, and grains was found to be significantly lower in individuals with higher stress (*p* < 0.02) [15, 16, 33–37]. Some studies assessed the intake of fast food, sweets, snacks, and energy drinks and found a direct association between these foods and perceived stress (p < 0.05) [15, 34, 37, 47, 48]. The consumption of meat and meat alternatives was measured in three studies and was inversely correlated with stress (p < 0.05) [16, 33, 35]. Mixed results were found in two studies that assessed alcohol intake: Gonzalez et al. [51] found that perceived stress was significantly associated with greater consumption of alcohol (p < 0.05) whereas Ng et al. [49] found no significant association (*p* = 0.4).

### Meta-analysis

Using the aforementioned methods for meta-analysis, 6 studies on diet quality were eligible for the meta-analysis [39–41, 43–45].

#### Assessment of heterogeneity

**Fig. 2:**
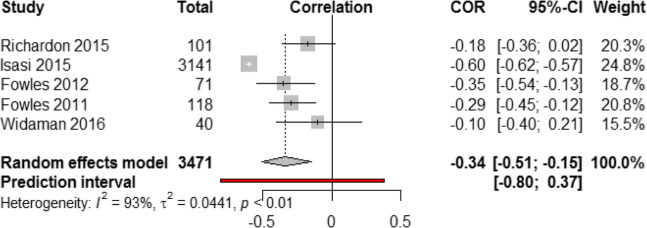
Association between stress and diet quality (five studies based on correlation coefficient “r” and converted β coefficients to “r”)

Outliers and influential analysis identified one outlier study [41]. Before removing this study from the analysis, the pooled effect was r = − 0.28 (95% CI [− 0.45; − 0.08], *p* value< 0.01). The overall effect size estimate (pooled correlation) was recalculated after removing this study and revealed a medium, negative, and very significant correlation (r = − 0.34, 95% CI [− 0.51; − 0.15], p value < 0.001) with 95% prediction interval of [− 0.80; 0.37]. These results (Fig. 2) suggest that a higher stress level was associated with poorer diet quality, and vice versa. The I^2^ heterogeneity measure in this analysis was substantial (93%), indicating significant variability across the studies (heterogeneity) and supporting the use of a random-effects model. Additionally, this conclusion was supported by Cochran’s *Q* test of heterogeneity which showed a very significant *P* value (< 0.0001).

Given the broad prediction interval in Fig. 2, which stretched well above zero, we cannot be 100% confident that the negative correlation between stress and diet quality found in this meta-analysis will be robust in every context.

#### Publication Bias

The funnel plot created was asymmetrical (Additional file 6). The asymmetry was mainly driven by one small size study [45] that has a large standard error and was shown in the bottom-right corner of the plot. This resembles a publication bias. Although this might occur due to chance, it might have also been comprised as a result of heterogeneity. The number of studies included in the meta-analysis was too small (5 studies) to test for significance of funnel plot asymmetry.

#### Sensitivity analysis

Trim-and-fill procedure identified three studies (Additional file 7) and assumed that initial results were underestimated due to publication bias. The true effect might be r = − 0.57 (95% CI [− 0.75; − 0.31], *p* value< 0.01) rather than r = − 0.34. Due to the assumed missing studies (small size studies reporting large effect sizes) and the small number of studies in this meta-analysis, the result of sensitivity analysis (r = − 0.57) is not considered a more valid estimate of the pooled correlation.

### Quality assessment

**Fig. 3:**
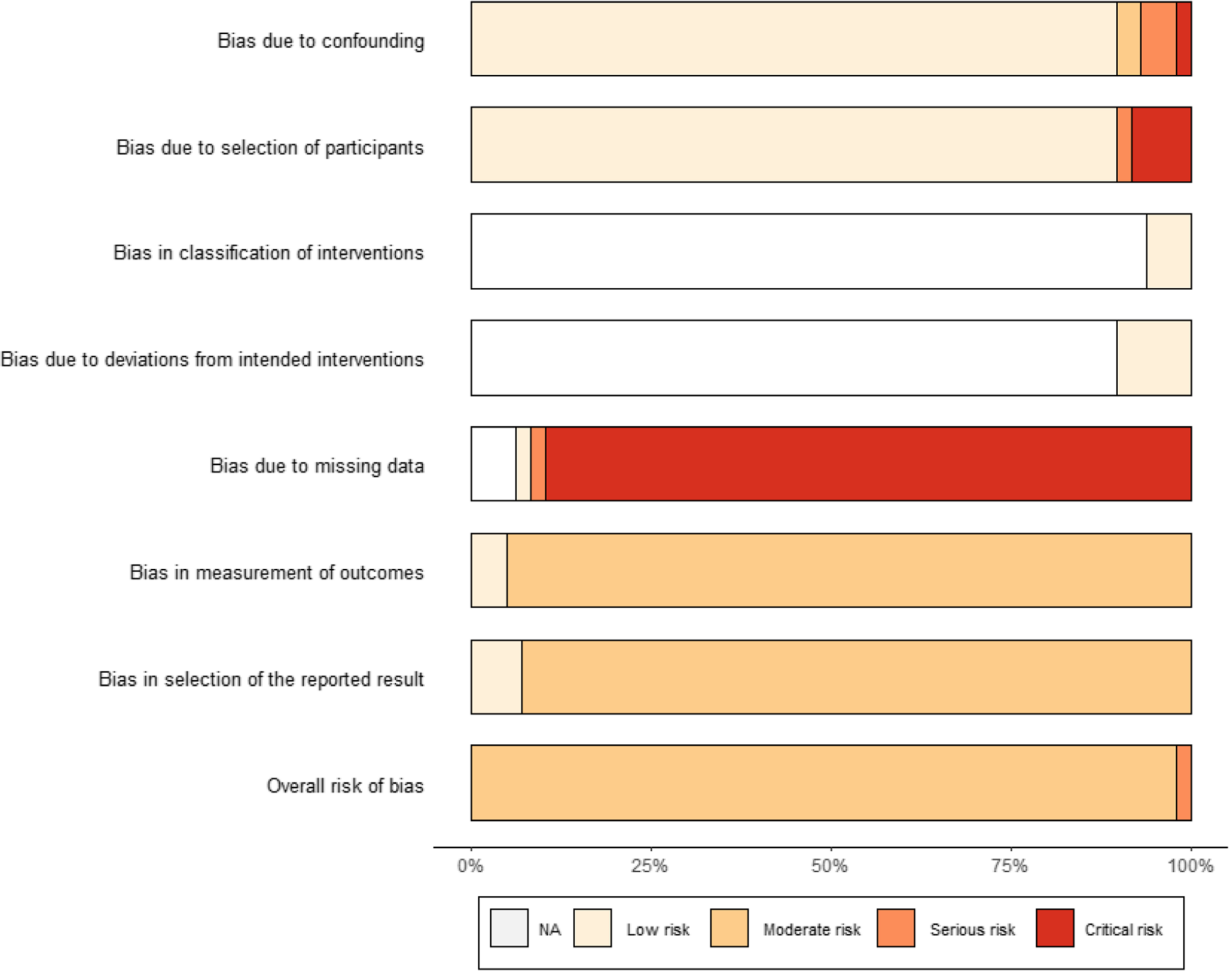
Weighed bar plot of the distribution of risk-of-bias judgments within each bias domain

Using “robvis” package, a weighed bar plot of the distribution of risk-of-bias judgments within each bias domain (Fig. 3) was generated to visualize the quality assessment performed for the 24 studies that were included in this systematic review.

Fig. 3 shows that most studies scored moderate with regards to bias in measurement of outcomes, selection of the reported results, and the overall risk of bias. More than 75% of studies had a critical risk of bias due to missing data. When it came to the bias due to confounding and selection of participants, around 90% of studies had a low risk, and most studies scored not available (NA) risk with regards to bias due to classification of interventions and deviations from intended interventions.

### Recruitment procedure

Recruitment procedures were very different among studies. In the eight studies on diet quality, three used data from participants enrolled in large cohorts from previous projects [10, 40, 42] while Fowles et al. [43, 44] recruited low income pregnant women in clinics using recruitment cards and forms (Table 1). The staff of a nutrition program helped Richardson et al. [39] identify women eligible for the study and the study staff asked them for their interest. Widaman et al. [45] recruited participants through advertisements on local newspapers, websites, and posted flyers while university students were recruited by distributing questionnaires during lectures [41]. Ethical approval was granted in seven studies and one study [40] did not give information regarding the ethical approval of the study.

Among the 16 studies of food intake and frequency of consumption, five studies used previous data of large cohort studies [33, 35, 37, 49, 52]. Eight studies recruited participants who were students through posters, flyers, or classroom visits at different university campuses [1, 15, 16, 34, 46–48, 51]. Participants of the three remaining studies were recruited differently; through community organizations [36, 50] or from staff of a large department store [49]. Three studies did not provide information regarding ethical approval [33, 49, 53], whereas all other thirteen studies mentioned that ethical approval was given prior to conducting the studies.

### Exposure: perceived stress

In four of the eight studies that assessed diet quality [10, 39–41], the Perceived Stress Scale (PSS) was used as a measure of psychological stress, whereas the other four studies used different scales such as: the General Health Questionnaire [42], the Prenatal Psychosocial Profile stress sub-scale [43, 44], and Wheaton Chronic Stress Inventory [45]. None of the studies used biomarkers of psychological stress (e.g. salivary cortisol) as a measure of the exposure.

All 16 studies that assessed food intake and frequency of consumption measured stress through self-reported measures: 10 studies used the Perceived Stress Scale [1, 15, 34, 37, 46–50, 53] and the six remaining studies used different other scales (Table 2).

### Dietary assessment

A variety of dietary instruments were used to assess habitual dietary intake in the eight studies that assessed diet quality. Three studies [10, 41, 42] used different Food Frequency Questionnaires (FFQs) to assess dietary intake (Table 1). The other five studies used 24-h dietary recalls for either: three days [43–45], two days [40], or one-to-two days [39].

With respect to diet quality, all studies used the a priori defined method (using diet indices) to derive the diet quality. A variety of diet quality indices were included: i) Alternate Healthy Eating Index [10, 40], ii) Healthy Eating Index [39, 45], iii) The Dietary Approach to Stop Hypertension (DASH) Diet Index [10, 42], iv) Dietary Quality Index- Pregnancy [43, 44], v) Dietary Guideline Adherence Index [41]. Interestingly, only one study combined three diet quality indices to measure diet quality [10], while all other studies used only one index. No study was found to assess diet quality via a posteriori approach i.e. to define diet patterns with statistical methods such as Factor Analysis.

There was also diversity in the tools used to assess food intake and frequency of consumption. Four of the 16 studies used food frequency questionnaires [15, 16, 34, 35], three used dietary recalls [36, 52, 53], another three used Block fat screener [1, 46, 49], two used alcohol intake frequency questions [49, 51], one used Block sodium screener [46], and one used weighed food records [50]. The remaining studies used different questions about food and beverages consumption (Table 1).

### Confounding factors

Table 1 indicates that seven of the eight studies of diet quality identified and corrected for socioeconomic status of participants as confounding factor. The exception was the study by Widaman et al. [45]. One study identified only age and educational level as means of socioeconomic status [44]. Three out of the eight studies did not assess the physical activity level of participants [39, 43, 44]. The anthropometric measures of participants were measured in all eight studies, either through BMI [10, 39, 41, 43–45] or both Waist Circumference and BMI [40, 42]. Smoking status was reported in three studies [42–44], marital status in five [10, 39, 42–44], and energy intake in three [10, 40, 45].

In the 16 studies of food intake and frequency of consumption, two studies did not identify or correct for confounding factors [15, 34]. All remaining studies identified socioeconomic status and demographic information of participants. Only five studies measured physical activity among participants [16, 35, 37, 49, 52]. BMI was reported in seven studies as a measure of adiposity [16, 35, 37, 46, 48, 50, 53] and only one study reported both waist circumference and BMI [52].

## Discussion

Our findings suggest that stress appears to impact diet negatively regardless of the various dietary outcomes measured among studies. Stress decreased diet quality and contributed to unhealthy dietary patterns, particularly high fat, fast food, sweets, and energy dense foods. In contrast stress lowered the intake of fruits, vegetables, fish and unsaturated oils.

The mixed results, especially in the eight studies on diet quality, highlights the disparity of evidence that exists in the literature regarding the association between stress and diet quality for the general population. In other populations, such as adolescents, perceived stress has been associated with poorer diet quality, measured through Diet Quality Index for Adolescents (DQI-A) (β = − 0.04, *p* < 0.01), [62]. An inverse association has been also reported in a systematic review with regards to mental health (including stress) and diet quality in children and adolescents [63] while Sims et al. [61] found no association between perceived stress and diet quality among female African American adults.

In almost all 16 studies on food intake and frequency of consumption included in our review, higher perceived stress was associated with an unhealthy eating pattern, characterised by increased consumption of sweets, fast food, fats and lower consumption of fruits and vegetables. This is in line with studies of other populations. Increased stress in female undergraduate students and peri-menopausal women has been linked with greater consumption of high calorie foods [64, 65]. Similarly, O’conner et al. [32] showed that daily stress was associated with a higher intake of high fat/sugar food and a reduced intake of fruits and vegetables in women. Wichianson et al. [30] found that stress was associated with unhealthy night-eating syndrome (NES) in a sample of 95 college students (β = 0.259, *p* < 0.05). Interestingly, one of the 16 studies on food intake and frequency of consumption found that stress was linked with decreased fat intake (along with all macro- and micro-nutrients) [52]. This contradicts the majority of studies in the field with only Torress et al. [23] finding an inverse association between stress and fat. Torres et al. [23] assessed daily record of stress and diet among male and female students and found that participants consumed less food and dietary fat when they were stressed. These conflicting results indicate that there might be inter-individual variation in response to stress.

The differences in results presented in Tables 1 and 2 must be interpreted with caution due to the challenges in assessing dietary intake. The eight studies on diet quality used different methods to collect dietary data: five studies used 24-h recalls [39, 40, 43–45] and mainly found negative association between stress and diet quality, while three studies [10, 41, 42] used food frequency questionnaires and found no association between stress and diet quality, which might explain the variance in the findings. Similarly, the 16 studies on food intake and frequency of consumption used food frequency questionnaires [15, 16, 34, 35], dietary recalls [36, 52, 53], block fat screener [1, 46, 49], and other different tools to assess dietary intake and found that stress was associated with the intake of unhealthy diet (higher fat, sweets, fast food, salt; lower fruits, vegetables, whole grains, and seafood). Although the use of food frequency questionnaires, 24-h dietary recalls, and the above-mentioned tools in nutrition epidemiology is quite common, measurement errors caused by self-reporting (under-reporting or over-reporting) of food intake occur leading to the manipulation of the expected associations. Furthermore, these dietary assessment methods might not be ideal for investigating the response to perceived stress; different methods such as ecologic momentary assessment, which aims to minimise recall bias, might be better in reporting dietary/behavioural responses to stress that take place in real time [40, 66].

Disparities exist between the two groups of studies in our review. Most of the 16 studies on food intake and frequency of consumption indicate that stress increases energy intake and food consumption [15, 36, 47, 48, 51–53]. In contrast, the majority of the eight studies on diet quality found no association between diet quality, which depends on food consumption, and stress. This can be explained mainly due to the diet quality indices used in the studies. Of the three studies that measured diet quality through the Healthy Eating Index (HEI) (including the Alternative HEI), two found no association between stress and diet quality [10, 39] and one found an inverse association [40]. However, out of the twelve scoring components of the HEI, nine will be scored higher if the intake of certain foods is higher which means that participants might have a higher energy and food consumption than they need and still score high on the HEI and have a higher diet quality. Moreover, the mixed findings could be related to the socioeconomic status of the participants as low socioeconomic populations tend to be more stressed than socially advantaged populations. A previous meta-analysis found that socioeconomically disadvantaged individuals had increased odds of being stressed and depressed (odds ratio = 1.81, *p* < 0.001) [67].

Two studies on diet quality were conducted among pregnant women [43, 44] and were included in the review since prenatal stress and diet are considered important for the intrauterine environment that affects several developmental outcomes [68–70]. The variation in diet quality of women during pregnancy has been associated with health outcomes of the fetus [71–76]. Similarly, maternal stress during conception is linked to disease risk and developmental outcomes of the fetus [68, 77–81]. More studies looking on diet and stress in this population and in the preconception stage are needed and should be conducted across different countries and with unified methodologies to allow comparison and confirm the stress/diet association.

### Strengths and limitations of the study

With diet quality and food intake in women of reproductive age being significant predictors of obesity and complications during pregnancy, the present systematic review adds to the body of knowledge by providing evidence on the role of psychological stress in manipulating diet quality. This will help in developing stress reducing strategies and guide future health care. The large sample size of most studies is a major strength of the present review. Another strength is restricting the sample to healthy women where studies with sample that had health conditions such as depression, metabolic diseases, and eating disorders were excluded, because these conditions might manipulate the diet quality and are considered significant confounding factors.

However, the 24 studies in the review are very heterogeneous in both participants that they recruited and the methods that they used, making pooling of these results challenging. Most of the eight studies on diet quality were conducted in USA and only two studies were conducted in the Middle East; no studies were conducted in Europe or Asia. This highlights the importance of conducting similar studies on diet quality among populations with different ethnicity and cultural backgrounds to confirm any possible differences. Another limitation is that in the 24 studies, stress was measured by self-reported stress scales and dietary intake was measured using 24-h recalls, food frequency questionnaires, or other self-reported questionnaires, which could lead to errors during dietary reporting and classification. A study measuring physiological markers of stress (such as serum or salivary cortisol) and biomarkers of dietary intake (such as urinary nitrogen, plasma vitamin C, and serum carotenoids) would provide stronger evidence. Moreover, differences in diet quality indices, dietary outcomes measured, and methodologies between the 24 studies made it difficult to compare the results of the studies. This issue has been highlighted by Mikolajczyk et al. [34] who recommended that research looking on stress and diet should be conducted across diverse population groups and amongst different countries which can enable the use of unified methodology and meaningful comparison of comparable outcomes. At present, it is challenging to compare results derived from studies conducted in single countries due to variation in methodologies and measures of diet and stress. The study design was a major limitation where studies were cross-sectional and longitudinal; hence, no causation or definitive conclusions can be drawn about the association between psychological stress and diet. A case-control study could provide more accurate evidence on the relationship between stress and diet. Including studies that are only in English language might be another limitation where evidence from studies published in other languages was not considered. Moreover, a prospective registration of this systematic review (for example on PROSPERO) was not done and this was also considered a limitation of this paper. The authors also declare that a thorough review/search of unpublished literature was not done, however the authors of unpublished papers were contacted and there were only 3 non-English abstracts found during the literature search.

## Conclusions

Studies exploring the association between stress and diet in women of reproductive age reported mixed results. This review adds to the current knowledge by highlighting the inverse association between stress and diet. However, there was substantial heterogeneity in both methods and outcomes, which made it difficult to pool the study results and draw a solid conclusion about the association between stress and diet quality/patterns. Studies of rigorous design and robust methodology are needed to determine the role of stress in manipulating the dietary patterns/quality of women of reproductive age. In particular, it is crucial to conduct studies in different countries, with larger number of participants, and with well-designed, unified and standardised methodologies.

Although some studies reported a significant association between stress and diet, this systematic review cannot determine causation of this association. At the clinical level, results from this systematic review, that showed inverse association between stress and healthy dietary patterns/quality in women of reproductive age, might be useful to implement stress coping strategies aimed at lowering stress levels and improving the quality of diet, and vice versa.

## Supplementary information

**Additional file 1: Table 1.** Search strategy.

**Additional file 2.**

**Additional file 3 Table 2**. Characteristics extracted from the 24 included studies: BS (Breakfast skippers), BE (Breakfast eaters), CS (Cross-Sectional), LG (Longitudinal), y (years), m (months), FFQ (Food Frequency Questionnaire, WFR (Weigh food record), SES (Socioeconomic status), PA (Physical Activity), AM (Anthropometric measures), − (not reported).

**Additional file 4: Table 3**. Data values extracted from the included eight studies on Diet Quality: β (Beta coefficients), r (correlation coefficient), OR (Odd Ratio).

**Additional file 5: Table 4**. Data values extracted from the included studies on food intake and frequency of consumption: ↑ (increase), ↓ (decrease), <= > (no association).

**Additional file 6.**

**Additional file 7.**

## Data Availability

Not applicable.

## Abbreviations

UK: United Kingdom
PRISMA: The Preferred Reporting Items for Systematic reviews and Meta-analysis Approach
PEO: Population, Exposure, and Outcome
AHEI: Alternate Healthy Eating Index
DASH: Dietary Approach to Stopping Hypertension
MDS: Mediterranean Diet Score
CASP: Critical Appraisal Skills Programme
PSS: Perceived Stress Scale
USA: United States of America
CI: Confidence Intervals
FFQ: Food Frequency Questionnaires
BMI: Body Mass Index
CS: Cross sectional
LG: Longitudinal
SES: Socioeconomic status
BS: Breakfast skippers
BE: Breakfast eaters
y: years
m: months
WFR: Weighed Food Records
PA: Physical activity
AM: Anthropometric measures
OR: Odd ratio
DQIA: Diet Quality Index for Adolescents
HEI: Healthy Eating Index
