# Supplementary material for: Perceived stress and diet quality in women of reproductive age: a systematic review and meta-analysis
Source: Nutr J. 2020 Aug 28;19:92. doi: 10.1186/s12937-020-00609-w (PMC7456060; doi:10.1186/s12937-020-00609-w)
Supplement: Supplementary file 1 — Additional file 1: Table 1. Search strategy. [file 12937_2020_609_MOESM1_ESM.docx]

| Diet | "diet* qualit*" or "diet* pattern*" or diet* or nutrition* or food intake* or food N5 consumption* or eating N5 habit* or eating behaviour* or " Mediterranean diet*" or "MDS" or "aMED" or priori or posteriori or Food or “Health* behaviour*” or energy N5 intake* or “nutrition* status” or “health* status” or eat* or appetite or “feeding behaviour*” |
| --- | --- |
| Stress | Stress* or anxiet* or anxious* or depress* or Psycholog* or distress* or emotion* |
| Women of reproductive age | wom#n or "childbearing age*" or "reproductive age*" or female* or premenopausal or "before pregnanc*” or “prior to conception” or “prior to pregnancy” or preconception |

**Table 1.** Search strategy
