# Supplementary material for: Perceived stress and diet quality in women of reproductive age: a systematic review and meta-analysis
Source: Nutr J. 2020 Aug 28;19:92. doi: 10.1186/s12937-020-00609-w (PMC7456060; doi:10.1186/s12937-020-00609-w)
Supplement: Supplementary file 2 — Additional file 2. [file 12937_2020_609_MOESM2_ESM.docx]

| \| **Reporting Criteria** \| \| --- \| | \| **Reported (Yes/No)** \| \| --- \| | \| **Reported on Page No.** \| \| --- \| |
| --- | --- | --- | --- | --- | --- |
| \| **Reporting of Background** \| \| --- \| |  |  |
| \| Problem definition \| \| --- \| | Yes | 2,3 |
| \| Hypothesis statement \| \| --- \| | Yes | 4 |
| \| Description of Study Outcome(s) \| \| --- \| | Yes | 4 |
| \| Type of exposure or intervention used \| \| --- \| | Yes | 4 |
| \| Type of study design used \| \| --- \| | Yes | 5 |
| \| Study population \| \| --- \| | Yes | 5 |
| \| **Reporting of Search Strategy** \| \| --- \| |  |  |
| \| Qualifications of searchers (eg, librarians and investigators) \| \| --- \| | Yes | 5 |
| \| Search strategy, including time period included in the synthesis and keywords \| \| --- \| | Yes | 4 |
| \| Effort to include all available studies, including contact with authors \| \| --- \| | Yes | 5 |
| \| Databases and registries searched \| \| --- \| | Yes | 4 |
| \| Search software used, name and version, including special features used  (eg, explosion) \| \| --- \| | Yes | 4 |
| \| Use of hand searching (eg, reference lists of obtained articles) \| \| --- \| | Yes | 5 |
| \| List of citations located and those excluded, including justification \| \| --- \| | Yes | 8 |
| \| Method for addressing articles published in languages other than English \| \| --- \| | Yes | 5 |
| \| Method of handling abstracts and unpublished studies \| \| --- \| | Yes | 5 |
| \| Description of any contact with authors \| \| --- \| | Yes | 5 |
| \| **Reporting of Methods** \| \| --- \| |  |  |
| \| Description of relevance or appropriateness of studies assembled for assessing the hypothesis to be tested \| \| --- \| | Yes | 5 |
| \| Rationale for the selection and coding of data (eg, sound clinical principles or convenience) \| \| --- \| | Yes | 6 |
| \| Documentation of how data were classified and coded (eg, multiple raters, blinding, and interrater reliability) \| \| --- \| | Yes | 6 |
| \| Assessment of confounding (eg, comparability of cases and controls in studies where appropriate \| \| --- \| | Yes | 6 |
| Assessment of study quality, including blinding of quality assessors; stratification or regression on possible predictors of study results | Yes | 6 |
| Assessment of heterogeneity | Yes | 7 |
| Description of statistical methods (eg, complete description of fixed or random effects models, justification of whether the chosen models account for predictors of study results, dose-response models, or cumulative meta-analysis) in sufficient detail to be replicated | Yes | 7 |
| Provision of appropriate tables and graphics | Yes | 5,8,11,12,13,16,17,18 |
| **Reporting of Results** |  |  |
| Table giving descriptive information for each study included | Yes | 16 |
| Results of sensitivity testing (eg, subgroup analysis) | Yes | 10 |
| Indication of statistical uncertainty of findings | Yes | 9,10 |
| **Reporting of Discussion** |  |  |
| Quantitative assessment of bias (eg, publication bias) | Yes | 12 |
| Justification for exclusion (eg, exclusion of non–English-language citations) | Yes | 21,22 |
| Assessment of quality of included studies | Yes | 13 |
| **Reporting of Conclusions** |  |  |
| Consideration of alternative explanations for observed results | Yes | 22 |
| Generalization of the conclusions (ie, appropriate for the data presented and within the domain of the literature review) | Yes | 22 |
| Guidelines for future research | Yes | 22 |
| Disclosure of funding source | Yes | 23 |
