# Supplementary material for: Perceived stress and diet quality in women of reproductive age: a systematic review and meta-analysis
Source: Nutr J. 2020 Aug 28;19:92. doi: 10.1186/s12937-020-00609-w (PMC7456060; doi:10.1186/s12937-020-00609-w)
Supplement: Supplementary file 3 — Additional file 3 Table 2. Characteristics extracted from the 24 included studies: BS (Breakfast skippers), BE (Breakfast eaters), CS (Cross-Sectional), LG (Longitudinal), y (years), m (months), FFQ (Food Frequency Questionnaire, WFR (Weigh food record), SES (Socioeconomic status), PA (Physical Activity), AM (Anthropometric measures), − (not reported). [file 12937_2020_609_MOESM3_ESM.docx]

| **Author, Year** | **Country** | **Age and Number of Participants** | **Study Design** | **Participants in Study** | **Dietary Assessment Tool** | **Confounding Factors Identified** |
| --- | --- | --- | --- | --- | --- | --- |
| *8 studies on Diet Quality* | | | | | | |
| *Richardson et al. 2015 [43]* | USA | 18-44 y, N=101 | CS | Women who had a child up to age 5 | 24-hour Dietary recalls | SES, AM |
| *Ferranti et al. 2013 [10]* | USA | Mean age 48 y, N=433 | LG (5 y follow up) | Male and female university and health center employees | FFQ | SES, PA, AM, |
| *Isasi et al. 2015 [42]* | USA | 18-74 y, N=3,141 | LG (9 m follow up) | Hispanic/Latino males and females | 24-hour Dietary recalls | SES, PA, AM |
| *El Ansari et al. 2015 [39]* | Egypt | 16-30 y, N=1,483 | CS | Undergraduate students males and females | FFQ | SES, PA, AM |
| *Valipour et al. 2017 [44]* | Iran | 28-45 years old, N= 2,134 | CS | General Adults | FFQ | SES, PA, AM |
| *Fowles et al. 2012 [41]* | USA | Mean age 24.7 y, N=71 | CS | Low income pregnant women | 24-hour Dietary recalls | SES, AM |
| *Fowles et al. 2011 [40]* | USA | Mean age 25 y, N=118 | CS | Low income pregnant women | 24-hour Dietary recalls | SES, AM |
| *Widaman et al. 2016 [45]* | USA | Mean Age 25.1,  N=35 (BS)  Mean Age 24.1,  N= 40 (BE) | CS | Female habitual breakfast eaters and breakfast skippers | 24-hour Dietary recalls | PA, AM |
| 16 studies on Food Intake and Frequency of Consumption | | | | | | |
| *Vidal et al. 2018 [1]* | Peru | Mean Age: 19 y, N= 272 | CS | Undergraduate students | Block fat screener | SES |
| *Nastaskin et al. 2015 [54]* | Canada | Mean age: 20 y, N=113 | CS | Students | Block fat screener/ Block sodium screener | SES, AM |
| *Pettit et al. 2011 [59]* | USA | 18-24 y, N=78 | CS | Undergraduate students | Energy drink intake questions | SES |
| *Mikolajczyk et al. 2009 [34]* | Germany, Poland, Bulgaria | Mean age: 20 y, N=1,201 | CS | Fist year undergraduate students | FFQ | - |
| *Errisuriz et al. 2016 [58]* | USA | Mean age: 18.9 y, N=433 | CS | Freshman students | Food and beverage frequency questions | SES, AM |
| *El Ansari et al. 2014 [15]* | UK | Mean age: 24.9 y, N=2,699 | CS | Students | FFQ | - |
| *Ng et al. 2003 [55]* | USA | Mean age: 40 y, N=6,620 | CS | Working adults | Block Fat Screener/ Alcohol frequency questions | SES, PA |
| *Barrington et al. 2012 [37]* | USA | 18-65 y, N=357 | CS | Working adults | Single-item question for fast food intake/ 5-A-Day fruit & vegetable assessment tool | SES, PA, AM |
| *Grossniklaus et al. 2010 [61]* | USA | Mean age: 41.3 y, N=64 | CS | Working adults | 3-day WFR | SES, AM |
| *Papier et al. 2015 [16]* | Australia | Mean Age 21.2 y, N=397 | CS | Students | FFQ | SES, PA, AM |
| *Roohafza et al. 2013 [35]* | Iran | Mean age: 38.4 - 39.5 y, N=9,549 | CS | General adults | FFQ | SES, PA, AM |
| *Gonzalez et al. 2013 [60]* | Puerto Rico | 21-30 y, N=186 | CS | First and second year students | Alcohol frequency questions | SES |
| *Tseng et al. 2011 [36]* | USA | Mean age 43.9 y, N= 426 | CS | Premenopausal women | 48- hour Dietary recalls | SES |
| *Hinote et al. 2009 [33]* | 8 post-Soviet  republics | >18 y, N=10,454 | CS | General adults | Questions about frequency of consumption | SES |
| *Hwang et al. 2010 [57]* | Korea | Mean age: 23.7 y, N=570 | CS | Vietnamese female marriage immigrants | 1-day Dietary recalls | SES, PA, AM |
| *Wardle et al. 2000 [56]* | UK | Mean Age: 36.29 y, N=58 | CS | Staff of a store | 24-hour Dietary recalls | SES, AM |

**Table 2.** Characteristics extracted from the 24 included studies: BS (Breakfast skippers), BE (Breakfast eaters), CS (Cross-Sectional), LG (Longitudinal), y (years), m (months), FFQ (Food Frequency Questionnaire, WFR (Weigh food record), SES (Socioeconomic status), PA (Physical Activity), AM (Anthropometric measures), - (not reported).
