# Supplementary material for: Perceived stress and diet quality in women of reproductive age: a systematic review and meta-analysis
Source: Nutr J. 2020 Aug 28;19:92. doi: 10.1186/s12937-020-00609-w (PMC7456060; doi:10.1186/s12937-020-00609-w)
Supplement: Supplementary file 4 — Additional file 4: Table 3. Data values extracted from the included eight studies on Diet Quality: β (Beta coefficients), r (correlation coefficient), OR (Odd Ratio). [file 12937_2020_609_MOESM4_ESM.docx]

| **Author, Year** | **Stress Assessment Tool** | **Diet Quality Index** | **Association between Stress and Diet Quality** | **β coefficient, r, or OR** |
| --- | --- | --- | --- | --- |
| *Richardson et al. 2015[43]* | - 14-item Perceived Stress Scale | - Healthy Eating Index 2010 | <=> | β = -0.18 (S.E 0.10, p=0.08) |
| *Ferranti et al. 2013 [10]* | - 14-item Perceived Stress Scale - Beck Depression Inventory II | - Alternate Healthy Eating Index - Mediterranean Diet Index - Dietary Approach o Stop Hypertension Index | <=> | Not reported |
| *Isasi et al. 2015 [42]* | - 10-item Perceived Stress Scale - 8-item Chronic stress burden | - Alternate Healthy Eating Index 2010 | ↓ | β = -0.61 (-1.18 to -0.03) |
| *El Ansari et al. 2015 [39]* | - 4-item Perceived Stress Scale | - Dietary Guideline Adherence Index | <=> | r= 0.00, p=0.98  β = 0.00 (-0.13 to 0.13) |
| *Valipour et al. 2017 [44]* | - 12-item General Health Questionnaire | - Dietary Approach o Stop Hypertension Index | <=> | OR: 1.02 (0.78-1.33) |
| *Fowles et al. 2012 [41]* | - Edinburgh Postnatal Depression Scale - Prenatal Psychosocial Profile-stress subscale | - Dietary Quality Index- Pregnancy | ↓ | r= -0.35, p is not reported |
| *Fowles et al. 2011 [40]* | - Edinburgh Postnatal Depression Scale - Prenatal Psychosocial Profile-stress subscale | - Dietary Quality Index- Pregnancy | ↓ | r= -0.293, p<0.01 |
| *Widaman et al. 2016 [45]* | - Wheaton Chronic Stress Inventory | - Healthy Eating Index 2010 | ↓ in breakfast skippers  <=> in breakfast eaters | Empty calories (r= -0.392, p= 0.027)  Empty calories (r= -0.104, p= 0.53) |

Table 3. Data values extracted from the included eight studies on Diet Quality: β (Beta coefficients), r (correlation coefficient), OR (Odd Ratio)
