# Supplementary material for: Perceived stress and diet quality in women of reproductive age: a systematic review and meta-analysis
Source: Nutr J. 2020 Aug 28;19:92. doi: 10.1186/s12937-020-00609-w (PMC7456060; doi:10.1186/s12937-020-00609-w)
Supplement: Supplementary file 5 — Additional file 5: Table 4. Data values extracted from the included studies on food intake and frequency of consumption: ↑ (increase), ↓ (decrease), <= > (no association). [file 12937_2020_609_MOESM5_ESM.docx]

| **Author, Year** | **Stress Assessment Tool** | **Association between Stress and the measured Food intake and frequency of consumption** | **Values** |
| --- | --- | --- | --- |
| *Vidal et al. 2018 [1]* | 14-item Perceived Stress Scale | ↑ Fat intake | p=0.005 |
| *Nastaskin et al. 2015 [54]* | 14-item Perceived Stress Scale | ↑ Fat intake  ↑Sodium intake | *r*=*.* 35, *p<0.*01  *r*=*.* 23, *p=0.*07 |
| *Pettit et al. 2011 [59]* | 14-item Perceived Stress Scale | ↑ Energy Drink intake | *r*=*.* 235, *p<0.*01 |
| *Mikolajczyk et al. 2009 [34]* | 14-item Perceived Stress Scale | ↑ Sweets, cookies, snacks, fast foods  ↓ Fruits/vegetables | p=0.03  p<0.01 |
| *Errisuriz et al. 2016 [58]* | Perceived stress single item scale (0-10) | ↑ Soda, coffee, energy drink, salty snack, sweet snack, frozen food, and fast food consumption | p<0.05 |
| *El Ansari et al. 2014 [15]* | 4-item Perceived Stress Scale | ↑ Sweets, cookies, snacks, fast food  ↓ Fruits and vegetables | P=0.017  P=0.002 |
| *Ng et al. 2003 [55]* | 4-item Perceived Stress Scale | ↑ High Fat diet  <=> Alcohol intake | p<0.01  p=0.4 |
| *Barrington et al. 2012 [37]* | 10-item Perceived Stress Scale | ↑ Fast food intake  ↓ Fruits and vegetables intake | z = 3.00, P= .003  z = −3.01, P = .003 |
| *Grossniklaus et al. 2010 [61]* | Perceived Stress Scale | <=> food and beverage intake | p>0.05 |
| *Papier et al. 2015 [16]* | Depression Anxiety Stress Scale (DASS) | ↑ processed foods  ↓ meat alternatives  ↓vegetables and fruits | p<0.01  p<0.05  p<0.01 |
| *Roohafza et al. 2013 [35]* | -A12-item General Health Questionnaire (GHQ-12) | ↑ Saturated oils  ↓ Unsaturated oils  ↓ Fruits  ↓ Vegetables  ↓ Meat  ↓ dairy products | p<0.01  p<0.01  p<0.01  p=0.02  p=0.03  p<0.01 |
| *Gonzalez et al. 2013 [60]* | Cognitivist Systemic Model Academic Stress scale | ↑ Alcohol intake | p<0.05 |
| *Tseng et al. 2011 [36]* | Migration–Acculturation Stressor Scale | ↑ Energy density  ↑ % energy from fat  ↓ total grams of grains  ↓ Overall grain intake | -(β= 0.002, p=0.04)  -(β=0.06, p= 0.05)  -(β=-11.3, p<0.0001)  -(β=-0.18, p=0.03) |
| *Hinote et al. 2009 [33]* | 12-item distress scale | ↓ Meat, fish, vegetables, fruits, animal fat | p<0.001 |
| *Hwang et al. 2010 [57]* | Psychological  Well-Being Index | - ↓ energy intake  - ↓ carbohydrates  - ↓ protein  - ↓ fat  - ↓ calcium  - ↓ vitamin A  - ↓ zinc  - ↓ thiamine  - ↓ riboflavin  - ↓ folate | -p=0.011  -p=0.004  -p=0.021  -p=0.021  -p=0.042  -p=0.039  -p=0.005  -p=0.006  -p=0.013  -p=0.004 |
| *Wardle et al. 2000 [56]* | 10-item Perceived Stress Scale | - ↑ energy intake  - ↑ saturated fats intake  - ↑ fat intake | -p<0.05  -p<0.01  -p<0.05 |

**Table 4.** Data values extracted from the included studies on food intake and frequency of consumption: ↑ (increase), ↓ (decrease), <=> (no association)
