# Supplementary figures and images for: Perceived stress and diet quality in women of reproductive age: a systematic review and meta-analysis
Source: Nutr J. 2020 Aug 28;19:92. doi: 10.1186/s12937-020-00609-w (PMC7456060; doi:10.1186/s12937-020-00609-w)

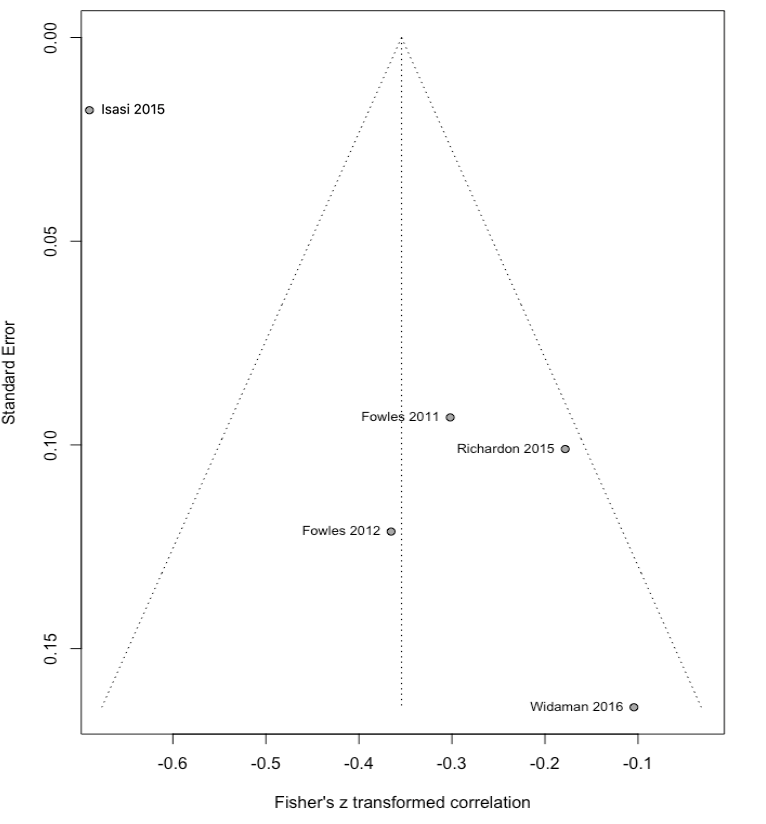

Supplement: Supplementary file 6 — Additional file 6. [file 12937_2020_609_MOESM6_ESM.png]

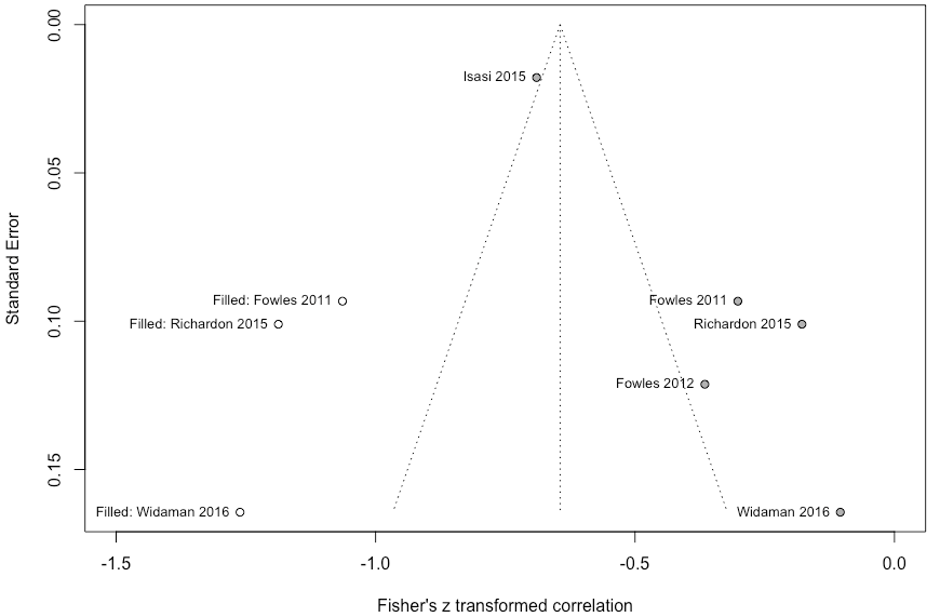

Supplement: Supplementary file 7 — Additional file 7. [file 12937_2020_609_MOESM7_ESM.png]
